# Ethical Principles Pertaining to the Care of People With Dementia: Protocol for a Qualitative Thematic Synthesis of Legal Documents

**DOI:** 10.2196/71490

**Published:** 2025-08-20

**Authors:** Daniel Sperling, Frederik Schou-Juul, Sigurd Lauridsen, Muhammad Asaduzzaman, Seda Guney, Dominika Kohanová, Vaitsa Giannouli, Corinna Porteri, Rodrigo Serrat, Ana Morais

**Affiliations:** 1 Department of Nursing Faculty of Social Welfare and Health Sciences University of Haifa Haifa Israel; 2 Faculty of Health Sciences, National Institute of Public Health University of Southern Denmark Odense Denmark; 3 Department of Community Medicine and Global Health Institute of Health and Society, Faculty of Medicine University of Oslo Oslo Norway; 4 School of Nursing Koç University Istanbul Turkey; 5 Department of Nursing Faculty of Social Sciences and Health Care Constantine the Philosopher University in Nitra Nitra Slovakia; 6 School of Social Sciences Hellenic Open University Patras Greece; 7 Bioethics Unit IRCCS Istituto Centro San Giovanni di Dio Fatebenefratelli Brescia Italy; 8 Cognition, Development, and Educational Psychology Department Faculty of Psychology University of Barcelona Barcelona Spain; 9 Department of Sports and Health School of Health and Human Development University of Évora Evora Portugal

**Keywords:** dementia care, legal frameworks, ethical principles, thematic synthesis, cross-country analysis

## Abstract

**Background:**

The global prevalence of dementia presents profound challenges for health care systems, societies, and legal structures worldwide. While the ethical dimensions of dementia care have been extensively discussed in the literature, limited research addresses how ethical principles are effectively operationalized within legal frameworks governing dementia care.

**Objective:**

We aim to explore how national, European, and international legal instruments integrate and translate ethical principles such as autonomy, dignity, beneficence, and justice into dementia care legal documents, including case law and legislation.

**Methods:**

This study will be conducted by a team of 24 researchers from 15 European countries, within a European Union (EU)–funded European Cooperation in Science and Technology Action on dementia care. The project applies a combined methodological approach, using qualitative thematic synthesis and a legal document review framework. Legal documents published between 2010 and 2025 will be searched and qualitatively analyzed at 3 levels: national, EU, and international, including legislation, case law, and authoritative legal literature explicitly addressing dementia care. Researchers from each country will conduct searches in national legal databases using predefined search terms. At the EU and international levels, databases such as Westlaw, Lexis+, the European Court of Human Rights, and the European Ombudsman will be consulted. Key information from each document will be collected using a standardized data extraction tool, focusing on ethical principles and frameworks. Data analysis will combine deductive and inductive approaches, allowing the identification of ethical principles and the emergence of new concepts discussed in the documents. Thematic analysis will follow a 3-stage process: line-by-line coding, grouping codes into descriptive themes, and developing analytical themes to address the research questions.

**Results:**

This study will identify and categorize ethical principles embedded in legal documents, analyzing their expression, interpretation, and variability across jurisdictions. The project is scheduled to be implemented throughout 2025. In January 2025, a pilot test of the data extraction tool was conducted. Between February 2025 and April 2025, a systematic search of legal documents meeting the inclusion criteria was performed, applying a structured 3-level search strategy. Data extraction and initial coding are planned from May 2025 to July 2025, followed by data analysis between September 2025 and October 2025, through iterative coding and collaborative discussion. The final research paper will be drafted in November 2025.

**Conclusions:**

This cross-country thematic synthesis will provide critical insights into how ethical principles guide dementia care and shape its discussion within legal systems. By systematically analyzing legal instruments through an ethical lens, this study aims to bridge the gap between ethical theory and legal practice, offering valuable guidance for future policy development. The findings will contribute to promoting ethically grounded and legally coherent frameworks for dementia care, safeguarding the rights and dignity of individuals living with dementia across diverse legal cultures.

**International Registered Report Identifier (IRRID):**

DERR1-10.2196/71490

## Introduction

### Background

The global prevalence of dementia is rising rapidly, presenting significant challenges for health care systems and societies worldwide. With the number of people living with dementia projected to triple by 2050 [1], there is an urgent need for legal frameworks that effectively address the ethical and practical concerns of dementia care. Ethical issues in dementia care typically arise when conflicting ethical principles or values generate uncertainty about the most appropriate course of action. While such dilemmas may have been less visible in earlier periods characterized by more paternalistic approaches to patient care, contemporary ethical and legal frameworks demand a more explicit and transparent consideration of ethical principles when supporting individuals living with dementia.

In caregiving contexts, these ethical challenges often emerge when individuals with dementia express preferences or make decisions that their caregivers perceive as imprudent or unsafe, for instance, the desire to continue driving despite clear evidence of cognitive decline. In legal or institutional settings, ethical concerns may involve the use of coercion in nursing homes, questions of testamentary capacity, or the individual’s ability to manage and dispose of personal property while experiencing progressive cognitive impairment and residing in a health care facility. Although extensive literature exists on the clinical and ethical dimensions of dementia, limited research has explored how legal frameworks operationalize ethical principles, particularly in the context of dementia care.

Legal frameworks, including binding legal instruments, such as international treaties, national legislation and case law, and nonbinding normative instruments, such as, guidelines determining legal relationships, rights, and duties or legal literature, play a crucial role in shaping dementia care, defining the rights and entitlements of individuals, and setting standards for the quality of care provided. These frameworks potentially have an impact on various aspects of life for people with dementia, including their right to receive appropriate care, promote their autonomy and decision-making capacity, maintain their dignity, and participate in society. The legal frameworks may be linked or otherwise include, either explicitly or implicitly, reference to or discussion of ethical principles. Under legal positivism, ethics, which is a subcategory in the discipline of philosophy, is separated and distinguished from law [2,3]. Yet, both ethics and law act as normative systems guiding human behavior. A natural law approach would argue that a law may serve as a formal expression of ethics, enforcing morality and promoting ethical values in society [4]. More generally, a law seeks to guide actions and decisions in many areas, including dementia care. It may establish exclusive rights, such as the right to receive proper care or make care decisions, and inalienable rights, such as the right to respect for human dignity [5]. Aside from and independent of the vast literature discussing ethics of dementia care [6-10], the law can be viewed as translating broad ethical principles into specific, actionable rules and regulations through the language of rights [11], which makes compliance with these principles mandatory. At the same time, by applying ethical principles, legal frameworks may gain more legitimacy and acceptance in their effect and power within society.

Research on the ethical principles underpinning, discussed, or referenced by legal frameworks is very limited, and, to the best of our knowledge, there are no studies systematically appraising ethical principles integrated into law within the field of dementia. Therefore, a thorough exploration and understanding of how and which ethical principles are integrated into laws governing dementia care is much needed and particularly vital in the clinical and policy contexts. Identifying the ethical principles and qualitatively understanding their role in legal regulation will make it possible to discern whether there is consistency between law and recognized ethical principles in dementia care.

### Objectives

This study aims to address this gap by providing a comprehensive cross-country thematic synthesis of the ways in which ethical principles play a role and are integrated into legal frameworks governing dementia care. Specifically, it seeks to comprehensively identify, explore, and categorize the spectrum of ethical principles related to dementia care as reflected in national and international legal frameworks and reported in various legal documents. In addition, this thematic synthesis aims to explore and analyze ethical conceptual frameworks such as the United Nations Charter on Human Rights or other national and international structures referred to in the legal documents. The study results will have the potential to inform policy and ultimately improve care for individuals with dementia.

## Methods

### Overview

This study aims to explore and examine the qualitative expression and role of ethical principles and ethical frameworks as reported in legal documents, which is described in the subsequent sections. This research project seeks to answer the following key questions:

1. How are ethical principles such as autonomy, dignity, and beneficence operationalized in national and international legal frameworks?
2. What are the most common ethical frameworks referenced in dementia care legislation, and how do they vary across different jurisdictions?
3. How can legal frameworks better harmonize ethical principles across countries to provide consistent care standards for individuals with dementia?

For the purposes of our study, we use the definitions mentioned in subsequent sections, which are also summarized in Textbox 1, along with key examples.

**A tentative summary of ethical and legal perspectives to explore.**

**Ethical principles**

- Autonomy
- Nonmaleficence
- Beneficence
- Justice
- Informed consent

**Ethical frameworks**

- Human rights
- Principlism ethics
- Justice based

**Legal documents**

- Legislation
- Case law
- Administrative guidelines
- Literature in legal journals

**Moral obligations and duties**

- Dignity
- Person centeredness
- Individuality, including political and democratic participation
- Privacy and confidentiality

### Principles (Including Ethical Principles)

In this study’s context, *principles* should be understood in the broadest possible sense. We define ethical principles as any generic normative claim, that is, claims about what one ought to do or what is good or bad and right or wrong. Thus, ethical principles include the classical principles of biomedical ethics, such as autonomy, nonmaleficence, beneficence, and justice, and encompass broader values, duties, moral obligations, and entitlements (such as dignity, person centeredness, individuality, political and democratic participation, privacy, and confidentiality).

### Ethical Frameworks

In this study’s context, ethical frameworks are defined as structured or systematic approaches or an organized set of principles that provide a foundation for assessing ethical issues. Ethical frameworks are meant to assist decision makers (eg, individuals or organizations) in making decisions about what is right or wrong and good or bad in various situations. Examples of such frameworks include the human rights ethical framework, principlism ethics, a justice-based ethical framework, etc.

### Legal Documents

For this study, we define legal documents as instruments regulating social aspects of life; establishing rules of behavior in society; disciplining the actions of public administration; resolving legal issues; developing business between people and companies; recording natural and social facts of legal relevance; and, in addition, recording, accumulating, sharing, and preserving theoretical legal knowledge [12]. Examples of these documents include legislation, case law, some administrative guidelines, and the literature published in legal journals.

### Design

This study will be conducted by a group of 24 researchers who are members and leaders in a European Union (EU)–funded COST Action on dementia care, representing 15 countries (Albania, Denmark, Estonia, Greece, Israel, Italy, Latvia, Lithuania, Macedonia, Norway, Portugal, Romania, Slovakia, Spain, and Turkey). This multinational team enhances the goals and robustness of the analysis by integrating diverse legal and cultural perspectives on dementia care. In addition, the team’s collective expertise in both legal and ethical dimensions ensures a proper understanding of how ethical principles are framed across different legal systems.

To achieve study goals, we will apply the qualitative thematic synthesis (QTS) method, developed by Thomas and Harden [13]. QTS is specifically designed for reviews or other qualitative reports, addressing questions about people’s perspectives and experiences that cannot be adequately assessed through traditional statistical analysis. This method is grounded in systematic review principles, combining rigorous search and qualitative coding strategies to formalize the identification and development of themes. It involves 5 consecutive steps. The first step involves *searching* for studies not necessarily to locate every relevant source but in accordance with the range of concepts found in the studies, their context, and whether they are in agreement or not. The second step involves the *quality assessment* of the findings, referring to the quality of reporting the studies’ aims, context, rationale, methods, and findings as well as the sufficiency of the strategies confirming the reliability and validity of the data collection tools and methods of analysis. It also refers to the assessment of the appropriateness of the methods at stake. The third step involves *extracting data*, usually including all the data presented under the Findings or Results sections of the studies. The fourth step involves *data synthesis* through coding text and developing themes, including free line-by-line coding and organization of *free codes* into related content areas, to construct descriptive themes. The fifth step includes *generating analytical themes*, that is, making judgments and going beyond the explored content. This last step has been identified as the defining characteristic of synthesis, as it involves conceptual innovation. The choice of QTS methodology for this study is highly appropriate, given the research aims and the types of sources of information to be reviewed, namely legal documents that provide narrations and discussions of ethical issues associated with dementia care. Moreover, QTS has been successfully applied in dementia and the ethics of dementia care [14-17].

Given the legal perspective of this study, we will also apply the framework proposed by Baude et al [18] for conducting a review of legal documents. This framework offers a 4-step process for conducting a review of legal documents. The first step involves *defining the exact question that the subsequent analysis is trying to answer*. Such a question should be precise and legalistic. This step also includes exploring what evidence is required for the question. The second step involves *defining the relevant sample of cases that were analyzed*. This step includes specifically mentioning what process was used to assemble the universe of cases or documents, stating any inclusion or exclusion criteria that were applied to the sample, and documenting the technology of the search process (databases, terms used, etc). The third step involves *stating how the cases in the sample were weighed in the analysis*. Examples of rules establishing proportional weight that are derived from legal doctrine include giving more weight to cases of greater precedential status, which are more recent, cited more frequently or written by more frequently cited judges, or include more analysis on the relevant topic. The fourth step involves *analyzing the sample and answering the question posed*. This step includes mentioning the criteria that were used to analyze the cases or documents, indicating how the cases were analyzed, and stating a conclusion that is supported by the analysis.

The framework by Baude et al [18] helps make legal claims through systematic demonstration of supporting evidence while also referring to the unique elements characterizing legal documents and ensures systematic identification, weighting, and analysis of legal texts. By combining QTS with a structured legal analysis framework, the study rigorously assesses the role of ethical principles in shaping dementia care legislation. This not only substantiates the claim but also allows for the assessment of its certainty or uncertainty, identifies potential disagreements in the referenced sources, reduces the risk of false statements, and helps minimize actual or potential bias, imprecision, subjectivity, and reliance on anecdotal evidence [12,18-20].

### Searching, Quality Assessment, and Data Extraction (Stages 1-3 Under QTS)

Our sample will include legal documents discussing ethical principles pertaining to the care of people with dementia following a 3-level systematic search strategy: national, EU, and international.

At the national level, researchers from each of the 15 participating countries will search national legal databases for relevant legal documents in their own languages. The documents must constitute either applicable law or rulings, court decisions, and case laws that pertain to dementia care.

At the EU level, the search will include European case law, notably published by the European Court of Human Rights, as well as petitions submitted to and legal decisions published by the European Ombudsman. In addition, the study will include EU regulations that pertain to dementia care. The documents must constitute either applicable law or case law, ensuring that the analysis captures both regulatory frameworks and judicial interpretations. All searched materials at the European level will be in English.

Finally, at the international level, the search will focus on three types of documents: (1) international treaties and covenants as well as rulings and court decisions published by the International Court of Justice or other similar international legal bodies, (2) legal texts from the United Nations and other international organizations that issue binding or influential guidelines and rulings on dementia care, and (3) literature published in international legal databases that can serve as acceptable legal sources and which interpret or apply legal rulings and discuss them. All searched materials at this level will be in the English language only. The inclusion and exclusion criteria for the legal documents are presented in Textbox 2.

Each researcher in the research team will conduct searches in their respective national legal databases according to a table of tasks allocated among members of the group. We will apply the first 2 stages suggested in the framework by Baude et al [18] to conduct a review for legal analysis. This stage will include the substages mentioned subsequently.

The search strategy will include the search terms (in the original language for the national level search and in English for the European and international levels) as presented in Textbox 3.

**Inclusion and exclusion criteria.**

**Inclusion criteria**

- The documents must explicitly but not exclusively mention dementia or conditions that pertain to dementia, for example, dementia care or the rights of people with dementia. The same text may also apply to other conditions, for example, intellectual disabilities.
- The documents must constitute applicable law (actual regulations), case law (rulings, decisions, etc), or the interpretation of it in international peer-reviewed legal literature indexed in legal databases.
- The documents must be published within the past 15 years (2010-2024) to ensure contemporaneity and relevance.

**Exclusion criteria**

- Gray literature that does not contain legal guidance, such as commentaries, opinions, or policy briefs that do not constitute law or case law, will be excluded.
- Peer-reviewed scientific articles published in nonlegal databases will be excluded.
- Legal documents that do not directly pertain to dementia will be excluded.

**Search strategy for dementia.**

**Keywords**

- Dement*
- Chronic brain disorder
- Alzheimer
- Neurocognitive impairment
- Cognitive impairment
- Neurocognitive decline
- Cognitive decline
- Neurocognitive loss
- Cognitive deterioration
- Neurocognitive disease
- Cognitive loss
- Neurocognitive dysfunction
- Cognitive disease
- Neurocognitive disorder
- Cognitive dysfunction
- Neurocognitive deficit
- Cognitive disorder
- Neurodegenerative impairment
- Cognitive deficit
- Neurodegenerative decline
- Memory impairment
- Neurocognitive loss
- Memory decline
- Neurodegenerative disease
- Memory loss
- Neurodegenerative dysfunction
- Memory disease
- Neurodegenerative disorder
- Memory dysfunction
- Neurodegenerative deficit
- Memory disorder
- Neurocognitive impairment
- Memory deficit
- Neurocognitive decline
- Neurocognitive loss

The final search strategy will make use of the following databases:

1. European Commission Library and e-Resources
2. Lexis+ (international)—search by country
3. Westlaw International Materials
4. European Court of Human Rights
5. European Ombudsman
6. National case law databases

We selected these databases for their unique characteristics and legal focus. These databases facilitate a comprehensive exploration of ethical principles and frameworks in dementia care that may be reported in legal documents. Texts that are not online or are not indexed under any of the aforementioned databases will not be analyzed.

The next stage involves reading the retrieved documents to ensure that they meet the inclusion and exclusion criteria. Relevant documents will be collected and cataloged for analysis. Researchers will use a standardized data extraction table to record key information from each document, including the title, publication date, jurisdiction, type of document (law, regulation, and case law), and a summary of the ethical principles mentioned (Multimedia Appendix 1). In addition, the charting form will be based on questions that specifically relate to searching legal documents following the framework by Baude et al [18]. It will allow the researchers to uniformly chart the legal documents for elements relevant to the research question. Moreover, the data charting process will focus on collecting both quantitative and qualitative elements in an effort to facilitate seamless analysis at a later stage. Any research member reviewing the text will be at liberty to add principles or frameworks to the data charting as long as they align with the aforementioned definitions. However, we will maintain detailed records of changes to the list of principles or frameworks, noting when and why a new principle is added or when an existing one is refined. This will enhance transparency and provide a clear audit of the decision-making process of our study. Moreover, we will continually reassess our data, using the updated charting form to check for extensiveness and accuracy in the data.

### Data Synthesis and Generating Analytical Themes (Stages 4-5 Under QTS)

Our data analysis process will include data synthesis through coding text and developing descriptive themes referring to the methodology by Thomas and Harden [13] and the framework by Baude et al [18] to conduct QTS.

Overall, to secure a rigorous analysis process, our analysis will use a combination of deductive and inductive approaches [21]. The deductive aspect will involve the use of a list of well-established ethical principles, which may serve as an inspiration for the research analysts (Multimedia Appendix 2). The list has been developed through iterations and in consultation with more general ethical literature discussing dementia, in particular, and care, in general, and following extensive work that has been done in the COST Action [22]. The inductive approach will enable us to identify ethical principles and frameworks without relying on any predetermined conceptual lens. Each analyst will be able to add more principles as the data analysis progresses after agreement on their wording and relevance to the research project among the research group.

The inductive approach will facilitate the identification of ethical principles that meet a predefined threshold of prevalence in our data. In addition, it will enable the reinterpretation of previously analyzed data, as outlined in the charting form. Our qualitative synthesis of the data will take the form of 3 intertwined stages [13]. The first stage is free line-by-line coding of the verbatim findings of the primary collected materials. In this stage, 2 researchers will independently code each line of the text according to its meaning and content at the European and international search levels and, when possible, at the national level. A list of codes will be prepared based on the translation of research principles or concepts put forth by each of the analysts and new principles or concepts added by analysts, when necessary. The second stage is the organization of “free codes” into related areas to construct “descriptive” themes. This process involves constant comparisons between and within codes for the purpose of exploring the similarities and dissimilarities among them. Following that, we will gradually group codes together in hierarchical order, adding and leaving out codes and categories until we establish agreed-upon tree-structured codes. The third stage is the development of “analytical” themes, including making judgment and going beyond the researched content. In this stage, which is regarded as the key characteristic of the synthesis [23], we will refer to the product of our analysis in its capacity to address the research aims and elucidate new concepts, understandings, and meanings concerning the role that ethical principles play in legal documents. Where possible, we will use ATLAS.ti or NVivo software (Lumivero) for the coding process as well as developing descriptive and analytical themes.

### Ethical Considerations

#### Human Participant Ethics Review Approvals or Exemptions

This study involves a QTS of publicly available legal and policy documents and does not involve direct interaction with human participants or include any patient data or personal information. Therefore, formal ethics committee approval was not required, in accordance with international and local guidelines for research involving publicly accessible secondary data.

#### Informed Consent

As no human participants were recruited or studied directly, informed consent was not applicable. All analyzed materials are legal and policy documents available in the public domain.

#### Privacy and Confidentiality

This study does not involve the collection or analysis of identifiable personal or sensitive data. All documents analyzed are identified and publicly available. Consequently, there are no concerns regarding privacy or confidentiality.

#### Compensation Details

No participants were recruited for this study, and no compensation was provided.

## Results

We expect to obtain the findings mentioned in the subsequent sections.

### Distribution of Ethical Principles

Our analysis will provide a rich description of the distribution of ethical principles of dementia care in legal documents. Such distribution will be made by (1) document type, including the frequency distribution of principles across document types (from most to least prevalent) and the qualitative explorations of each of these principles by document type; (2) chronological trends, identifying evolutionary patterns in the discussion and prioritization of ethical principles; and (3) target population, pointing to the differences, if any, of the discussions of ethical principles in documents focusing on persons with dementia compared to formal and family caregivers.

### Principle Conflict Identification

We will describe the distribution of cases that address conflicts between ethical principles, for example, autonomy versus beneficence, or professional obligations versus family preferences. Such conflicts will be analyzed by document type, chronological trends, and target population.

### Qualitative Analysis of Key Principles

The key principles that will be found across the documents will be qualitatively analyzed by describing and discussing their expected codes and contexts*.* For example, we believe that concerning the principle of personal autonomy, expected codes may include self-determination, informed consent, capacity assessment, supported decision-making, and advance directives. The expected context of these principles is likely to be framed differently based on the document type, with legal documents emphasizing procedural aspects of autonomy, while clinical literature may focus on relational autonomy concepts.

### Resolution Frameworks

Legal documents that will include tensions or conflicts between ethical principles will also be described according to prevalent theories by which they will be resolved, for example, principlism, care ethics, rights-based approaches, consequentialism, casuistry (case-based reasoning), and the like.

### Qualitative Analysis of Resolution Strategies

Legal documents that will include tensions or conflicts between ethical principles will also be qualitatively analyzed. Expected codes include balancing tests, best interests assessments, substituted judgment, procedural safeguards, shared decision-making, ethics consultation, and proportionality analysis.

### Judicial Analysis

For case law only, we also expect to have additional results pertaining to the (1) significance of ethical principles, referring to the question of how much of a central role ethical principles play in the judicial decisions addressing relevant care situations; (2) judicial disagreement**,** examining the proportion of cases that will show split opinions or dissents specifically related to the interpretation or prioritization of ethical principles; (3) normative hierarchy analysis, assuming that principles with strongest legal protection (legislation and constitutional rights) will typically prevail over principles derived from lower-ranked sources, such as case law or policy documents; and (4) citation analysis, including citation rates, that is, the proportion of documents that include citations to other ethical sources or precedents, the ethical principles receiving most citations, and citation sources of ethical principles**.**

### Unexpected Data Patterns and Proposed Timeline

Along these expected results, we will remain alert to unexpected patterns in the data, such as the emergence of newer ethical principles not traditionally emphasized in bioethics literature, significant jurisdictional differences in ethical approaches, or novel resolution frameworks not anticipated in the initial coding structure.

Given the changing and updating nature of law and legal materials, we propose the timeline for this project:

- January 2025; a pilot test of the data extraction tool was conducted.
- February to April 2025; a systematic search of legal documents meeting the inclusion criteria was performed, applying a structured three-level search strategy.
- May to July 2025; data extraction and initial coding are planned.
- September to October 2025; data analysis will be conducted through iterative coding and collaborative discussion.
- November 2025; the final research paper will be drafted.

## Discussion

### Anticipated Findings

This study seeks to advance our understanding of how ethical principles are embedded within legal frameworks relating to dementia care, within the national legal systems of the participating countries as well as in wider EU and international legal frameworks. We anticipate that our analysis will reveal the extent to which ethical principles, such as autonomy, dignity, beneficence, and justice, are expressed, interpreted, or contested within binding legal texts and normative legal instruments. Moreover, by examining legal materials from 15 countries alongside the European and international instruments, we expect to identify jurisdictional variability and differing interpretations of ethical principles. Provided that we identify such variability in our analysis, this will be reported in the final study.

In addition, we expect to find both direct and indirect references to ethical principles, which may be framed through rights-based discourses, procedural safeguards, or institutional responsibilities. Through this, this study will contribute to a better understanding of the legal translation of ethical norms and the degree to which such norms are enforceable, aspirational, or symbolic in national and supranational regulatory contexts.

While substantial literature exists on the ethical aspects of dementia care, to the best of our knowledge, no studies have explored their concrete integration in legal instruments. The 2009 Nuffield Council on Bioethics report on dementia [24] remains a foundational document, emphasizing the need to uphold values such as respect for autonomy, social inclusion, and balancing risks with potential benefits in dementia-related decision-making. However, the report stops short of exploring how these values are formalized and protected in law across different jurisdictions. Similarly, Strech et al [10] offered a systematic review of ethical issues in dementia care, identifying a broad range of dilemmas, particularly regarding consent, risk management, and personhood, but without addressing the legal codification of these challenges. Finally, Johnson and Karlawish [25] reviewed emerging ethical issues in dementia care but did not examine how these issues and principles are addressed within legal frameworks.

More recent works [7] have focused on specific areas such as supported decision-making and legal tools for addressing manifestations of dementia, yet there is no comparative, systematic thematic analysis of legal instruments through an ethical lens.

Our study builds on this gap by applying legal and philosophical frameworks in tandem to assess how ethical principles function within regulatory texts, which allows us to explore not only the principles that are present but also how they are interpreted and potentially contested across diverse legal cultures.

### Strengths and Limitations

A core strength of our project is its multidisciplinary and multinational design, combining expertise in law, ethics, health policy, and dementia care across 15 countries. This enables robust triangulation of perspectives and enhances cultural and legal contextualization. Methodologically, the integration of QTS with a structured legal review process [18] provides a unique and replicable approach to doctrinal legal analysis grounded in qualitative rigor. Screening and selecting the documents for this thematic synthesis will be systematically conducted by a multidisciplinary team with backgrounds in law, philosophy, psychology, nursing, medicine, and gerontology. Our aim is to promote rigor and replicability in the document screening and selection processes, adhering to the following three-step systematic approach:

1. Initial abstract or ratio decidendi review (when applicable)—at least 2 research group members will independently assess abstracts for study screening in the European and international search levels, and when possible, at the national level as well.
2. Reviewer meetings—regular meetings will be held during the study screening and selection process to discuss and resolve any challenges or uncertainties regarding study selection and ensure relevance to the study objectives.
3. Full document review and analysis—Following the abstract or ratio review, analysts will independently assess the full documents of selected abstracts for final inclusion. Any uncertainties among the researchers will be resolved at team meetings. Where no abstract or ratio decidendi appears, documents will be reviewed in full at the first stage.

However, both inherent and anticipated limitations must be acknowledged. First, the analysis will depend on the researchers’ linguistic and legal familiarity with national documents, which may introduce interpretive variation. Moreover, the multidisciplinary composition of the research team, while a major strength, also presents a limitation; not all team members may have previous experience working with legal texts, and some may not be formally trained in identifying ethical principles, particularly from a philosophical or normative standpoint.

To mitigate these risks and enhance quality assessment, we will consider both the limitations of our reporting, given the great variety and subjectivity of the assessment tools and the fluid content, which is subject of the synthesis, and explain the quality assessment process, including the criteria by which cases have been excluded from our synthesis [26]. Moreover, all coding and thematic development will involve structured peer debriefing, collaborative consensus building to the greatest extent possible, and the use of standardized charting forms. These forms will be accompanied by detailed guidance on both their use and the process of identifying and interpreting ethical principles within legal materials. In addition, our report will follow and apply the 21 items put forward in the Enhancing Transparency in Reporting the Synthesis of Qualitative Research statement, referring to 5 main domains: introduction, methods and methodology, literature search and selection, appraisal, and synthesis of findings [27].

### Conclusions

This study will provide a comprehensive, cross-country thematic synthesis of how ethical principles, such as autonomy, dignity, beneficence, and justice, are embedded, interpreted, and sometimes contested within legal frameworks governing dementia care. By systematically analyzing legal instruments across national, European, and international contexts through an ethical lens, this study highlights the extent to which these principles are operationalized in law. The integration of ethical norms into legal frameworks is crucial not only for ensuring medically appropriate care but also for safeguarding the rights and dignity of individuals living with dementia. This study’s multidisciplinary approach, rigorous methodology, and focus on transparency contribute to bridging the gap between ethical theory and legal practice. Its findings may have important implications for policy makers, legal professionals, and health care providers, offering guidance for the development of more ethically grounded and legally coherent regulatory frameworks.

## Appendix

Multimedia Appendix 1 Data extraction chart.

Multimedia Appendix 2 List of ethical principles.

## Abbreviations

EU: European Union
QTS: qualitative thematic synthesis

## Data Availability

The datasets generated or analyzed during this study are available from the corresponding author on reasonable request.
