# Correction: Ethical Principles Pertaining to the Care of People With Dementia: Protocol for a Qualitative Thematic Synthesis of Legal Documents

**DOI:** 10.2196/87273

**Published:** 2025-11-28

**Authors:** Daniel Sperling, Frederik Schou-Juul, Sigurd Lauridsen, Muhammad Asaduzzaman, Seda Guney, Dominika Kohanová, Vaitsa Giannouli, Corinna Porteri, Rodrigo Serrat, Ana Morais

**Affiliations:** 1 Department of Nursing Faculty of Social Welfare and Health Sciences University of Haifa Haifa Israel; 2 Faculty of Health Sciences, National Institute of Public Health University of Southern Denmark Odense Denmark; 3 Department of Community Medicine and Global Health Institute of Health and Society, Faculty of Medicine University of Oslo Oslo Norway; 4 School of Nursing Koç University Istanbul Turkey; 5 Department of Nursing Faculty of Social Sciences and Health Care Constantine the Philosopher University in Nitra Nitra Slovakia; 6 School of Social Sciences Hellenic Open University Patras Greece; 7 Bioethics Unit IRCCS Istituto Centro San Giovanni di Dio Fatebenefratelli Brescia Italy; 8 Cognition, Development, and Educational Psychology Department Faculty of Psychology University of Barcelona Barcelona Spain; 9 Department of Sports and Health School of Health and Human Development University of Évora Evora Portugal

In “Ethical Principles Pertaining to the Care of People With Dementia: Protocol for a Qualitative Thematic Synthesis of Legal Documents” [1] the authors noticed that the Acknowledgments statement had been inadvertently omitted, and have now added the following:

This article is based upon the work from COST Action EDEM (Ethics in Dementia; CA21137), supported by COST (European Cooperation in Science and Technology).

The correction will appear in the online version of the paper on the JMIR Publications website, together with the publication of this correction notice. Because this was made after submission to PubMed, PubMed Central, and other full-text repositories, the corrected article has also been resubmitted to those repositories.
