# Supplementary material for: Ethical Principles Pertaining to the Care of People With Dementia: Protocol for a Qualitative Thematic Synthesis of Legal Documents
Source: JMIR Res Protoc. 2025 Aug 20;14:e71490. doi: 10.2196/71490 (PMC12409177; doi:10.2196/71490)
Supplement: Multimedia Appendix 1 [file resprot_v14i1e71490_app1.pdf]

Country	Type of document	Article / Document reference	Year	Target Population (PwD; Formal / Informal Caregivers; etc)	The document discussed ethical principles regarding dementia care: Y/N

What ethical principles were discussed?	Principle 1	Principle 2	Principle 3

Please provide an example of how this ethical principle/s is articulated	How many times the document includes reference to these principles?	Did the document refer to a conflict between ethical principles? Y/N

If yes, between which principles?	How has the document resolved the conflict?	If by reference to a theory/framework, how many times does the document refer to such a theory/framework?

(for case law only) Did the discussion of the ethical principles/conflict have significant role in the overall document/decision?	(for case law only) Was there a split/disagreement in how the document referred to ethical principles?	(for case law only) Was there a disagreement/split in how the document referred to conflict between ethical principles?

Does the document have greater precedential status?<br>1- legislation<br>2- supreme court<br>3- district court<br>4- lower courts<br>5- literature<br>6 – grey literature, guidelines, administrative etc.	Was the legal document cited?<br>If so, how many times?	If so, where?	Other comments
