# Supplementary material for: Ethical Principles Pertaining to the Care of People With Dementia: Protocol for a Qualitative Thematic Synthesis of Legal Documents
Source: JMIR Res Protoc. 2025 Aug 20;14:e71490. doi: 10.2196/71490 (PMC12409177; doi:10.2196/71490)
Supplement: Multimedia Appendix 2 [file resprot_v14i1e71490_app2.docx]

List of Ethical Principles

| **Respect for human dignity** |
| --- |
| **Person-Centeredness and Individuality** |
| **Preservation of the body and its proper appearance** |
| **Personal autonomy and freedom of choice** |
| **Maintaining privacy and confidentiality** |
| **Possession of things and property** |
| **Maintaining familial ties and association** |
| **Political and democratic participation** |
| **Social engagement, including engagement with meaningful activities, and experiences** |
| **De-stigmatization** |
| **Inclusive and adapted living environments** |
| **Evidence-based practice and care** |
| **Holistic and synergetic approach to care** |
|  |
